# Is Nav1.8-specific inhibition the only path? Preferential Nav blockers offer a compelling alternative

**DOI:** 10.1073/pnas.2519744122

**Published:** 2025-09-10

**Authors:** Bingbing Xiang, Chaoyi Deng, Han Yang, Wensheng Zhang

**Affiliations:** ^a^Department of Anesthesiology, West China Hospital, Sichuan University, Chengdu 610041, China; ^b^Laboratory of Anesthesia and Critical Care Medicine, National-Local Joint Engineering Research Centre of Translational Medicine of Anesthesiology, West China Hospital, Sichuan University, Chengdu 610041, China

We have read with interest the study by Stewart et al. (1) and concur with their conclusion that selective inhibition of Nav1.8 alone is insufficient to robustly suppress peripheral nociceptive signaling. NaV1.8—which conducts much of the current underlying the action potential signal—was supported by weaker genetic validation (2).

VX-548, a Nav1.8 inhibitor, recently reported results from a Phase II trial in lumbosacral radiculopathy: While demonstrating statistically significant pain reduction, its efficacy was marginal compared to placebo, sparking concerns about its clinical viability. Moreover, in two prior Phase II trials, VX-548 reduced pain vs placebo over 48 h only at the highest dose, with no lower-dose efficacy (3). This is striking given its exceptional Nav1.8 selectivity (30,000 to 40,000-fold over other Nav subtypes). The lack of lower-dose effect despite such selectivity raises a key question: Does the highest dose’s efficacy stem from off-target effects rather than specific Nav1.8 inhibition? At these concentrations, VX-548 could plausibly cause nonselective Nav blockade—similar to intravenous lidocaine’s mechanism (4, 5)—undermining the premise that its activity is solely from Nav1.8 inhibition. More recently, two Phase III trials (6) reported that VX-548 significantly reduced pain vs placebo, with 61.7% of treated patients rating overall response as “good” or “excellent” at 48 h (vs. 53.2% for placebo). However, this magnitude of benefit is clearly insufficient for achieving robust analgesia.

This pattern aligns with the insights from Stewart et al. (1): Nav1.8 inhibition reduces neuronal excitability but incompletely, failing to fully block firing in pain-transmitting dorsal root ganglion neurons. Collectively, these findings underscore a fundamental limitation of selective Nav1.7 or Nav1.8 inhibition: Nociceptive signaling relies on a redundant network of Nav channels, making single-target blockade insufficient for robust analgesia. Preferential blockers, which modulate multiple relevant Nav subtypes while sparing off-target channels, may thus represent a more promising strategy to overcome this translational hurdle.

Critical questions persist: Is complete suppression of all pain-transmitting neurons requisite for effective analgesia? Must sustained firing be entirely eliminated (i.e., no residual action potentials) to achieve meaningful pain relief? Notably, these uncertainties stand in contrast to a clear benchmark: Local anesthetics, which broadly block Nav channels, reliably abolish action potentials in nociceptive neurons and thereby fully interrupt pain signaling. This observation points to a provocative alternative: Preferential Nav channel inhibitors, designed to cotarget multiple pain-relevant subtypes (e.g., Nav1.7, Nav1.8, and Nav1.9), may offer a more effective strategy. As Waxman al. (7) argue, the limited analgesic efficacy of selective Nav1.8 inhibition likely reflects the redundancy of ion channel contributions to nociceptive signaling—with multiple channels and mechanisms converging to drive neuronal excitability.

Against this backdrop, the path forward warrants cautious optimism. The persistent challenges of subtype-selective Nav inhibitors highlight the complexity of pain signaling networks, but they also underscore the potential of preferential blockers to harness coordinated Nav channel modulation. Such strategies, balancing selectivity for pain-relevant subtypes with broad enough coverage to overcome redundancy, may finally bridge the gap between preclinical promise and clinical impact in nonopioid analgesia.
